# Amino Acid Medical Foods Provide a High Dietary Acid Load and Increase Urinary Excretion of Renal Net Acid, Calcium, and Magnesium Compared with Glycomacropeptide Medical Foods in Phenylketonuria

**DOI:** 10.1155/2017/1909101

**Published:** 2017-05-04

**Authors:** Bridget M. Stroup, Emily A. Sawin, Sangita G. Murali, Neil Binkley, Karen E. Hansen, Denise M. Ney

**Affiliations:** ^1^Department of Nutritional Sciences, University of Wisconsin-Madison, Madison, WI, USA; ^2^Department of Medicine, Divisions of Endocrinology and Geriatrics, University of Wisconsin School of Medicine and Public Health, Madison, WI, USA; ^3^Department of Medicine, Divisions of Rheumatology and Endocrinology, University of Wisconsin School of Medicine and Public Health, Madison, WI, USA

## Abstract

*Background*. Skeletal fragility is a complication of phenylketonuria (PKU). A diet containing amino acids compared with glycomacropeptide reduces bone size and strength in mice.* Objective*. We tested the hypothesis that amino acid medical foods (AA-MF) provide a high dietary acid load, subsequently increasing urinary excretion of renal net acid, calcium, and magnesium, compared to glycomacropeptide medical foods (GMP-MF).* Design*. In a crossover design, 8 participants with PKU (16–35 y) provided food records and 24-hr urine samples after consuming a low-Phe diet in combination with AA-MF and GMP-MF for 1–3 wks. We calculated potential renal acid load (PRAL) of AA-MF and GMP-MF and determined bone mineral density (BMD) measurements using dual X-ray absorptiometry.* Results*. AA-MF provided 1.5–2.5-fold higher PRAL and resulted in 3-fold greater renal net acid excretion compared to GMP-MF (*p* = 0.002). Dietary protein, calcium, and magnesium intake were similar. GMP-MF significantly reduced urinary excretion of calcium by 40% (*p* = 0.012) and magnesium by 30% (*p* = 0.029). Two participants had low BMD-for-age and trabecular bone scores, indicating microarchitectural degradation. Urinary calcium with AA-MF negatively correlated with L1–L4 BMD.* Conclusion*. Compared to GMP-MF, AA-MF increase dietary acid load, subsequently increasing urinary calcium and magnesium excretion, and likely contributing to skeletal fragility in PKU. The trial was registered at clinicaltrials.gov as NCT01428258.

## 1. Introduction

PKU (PKU; OMIM 261600) is an inherited metabolic disorder characterized by high Phe concentrations in blood due to mutations in the gene which encodes phenylalanine hydroxylase (PAH; EC 1.14.16.1). PAH catalyzes the hepatic conversion of Phe to Tyr using tetrahydrobiopterin as a cofactor [1]. Untreated PKU causes Phe to accumulate in the brain resulting in profound cognitive impairment [2]. The primary therapy for PKU is lifelong adherence to a low-Phe diet that limits Phe intake from natural foods and supplements with AA-based medical foods (AA-MF), often referred to as protein substitutes. AA-MF are consumed alone or in combination with administration of the PAH cofactor (sapropterin dihydrochloride) [3].

Skeletal fragility, characterized by low bone mineral density (BMD) and increased fracture risk, has emerged as a poorly understood chronic complication of PKU managed with AA-MF [4–6]. There is no consensus on the incidence, etiology, implications, and risk factors for low BMD in PKU. Low BMD is reported in 40–50% of adults with PKU [6]. Likewise, 33% of children with PKU have BMD at least two standard deviations (*Z*-score ≤ −2) below the expected range for age [7]. The etiology of skeletal fragility in PKU is unknown and current knowledge reflects cross-sectional studies. Our murine data established a PKU bone phenotype characterized by decreased BMD and defective bone biomechanical performance, which was worsened by a low-Phe amino acid (AA) diet in association with increased renal net acid and calcium excretion and increased renal mass [8–10]. Strikingly, both wild type and PKU (*Pah*^enu2^) mice fed a low-Phe glycomacropeptide diet, which provides a low dietary acid load relative to the AA diet, develop stronger bones and lower renal mass compared with mice fed the AA diet [8, 9]. Consistent with these preclinical data, researchers have reported acid-base disturbances [11] and impaired kidney function [12] when individuals with PKU consumed AAs as the primary source of dietary protein.

Chronic compensation for a high dietary acid load is acknowledged to increase bone resorption and urinary calcium excretion leading to lower BMD and increased fracture risk [13–16]. We tested the hypothesis that AA-MF, used in the nutritional management of PKU, provide a high dietary acid load, resulting in increased urinary excretion of renal net acid, calcium, and magnesium, compared with GMP-MF. Participants in this pilot study include a subset of 8 of the 30 individuals with PKU who completed our controlled clinical trial [17].

## 2. Methods

### 2.1. Experimental Approach

We determined the potential renal acid load (PRAL) provided by commercially available AA-MF and GMP-MF in 2013-2014. Ten AA-MF and 3 GMP-MF (*n* = 2-3 per medical food) were analyzed for mineral and AA content and PRAL was calculated to predict the dietary acid load using the following equation: PRAL = (2 × (0.00503 × mg Met/d)) + (2 × (0.0062 × mg Cys/d)) + (0.037 × mg phosphorus/d) + (0.0268 × mg chloride/d) − (0.021 × mg potassium/d) − (0.026 × mg magnesium/d) − (0.013 × mg calcium/d) − (0.0413 × mg sodium/d) [18, 19]. We assessed how medical foods, differing in dietary acid load, affected excretion of renal net acid and bone-related biomarkers. Additionally, we measured serum 25-hydroxyvitamin D, 1,25-dihydroxyvitamin D, and bone turnover markers and plasma calcium and cytokine concentrations in the majority of the 30 participants in the full clinical trial at the final study visit; these data are reported herein for the first time [17].

### 2.2. Study Design and Protocol to Assess Renal Net Acid and Mineral Excretion

We conducted a 2-stage, controlled, crossover intervention pilot study that compared urinary biomarker excretion in 8 free-living participants with early treated PKU following a low-Phe diet combined with either AA-MF or GMP-MF. Participants were recruited from those already enrolled in the primary clinical trial at the University of Wisconsin-Madison site. To control for potential carry-over effects of GMP on calcium absorption, seven of 8 participants completed the protocol with the AA-MF treatment first (their usual diet), followed by the GMP-MF treatment. Additional inclusion criteria for enrollment [17] included (1) consumption of a high-PRAL AA-MF and (2) ability to transport two 24-hour urine collections to the study center. The University of Wisconsin-Madison Health Sciences review board approved the study protocol. All participants provided written informed consent. The trial was registered at www.clinicaltrials.gov as NCT01428258.

Participants consumed a low-Phe diet with high-PRAL AA-MF and low-PRAL GMP-MF for 1–3 wks. Participants provided a 24-h urine collection within the last 24–36 hours of the participants' study protocol, two to three consecutive 24-h food records before and during the period of urine collection, and a fasting dried blood spot for determination of Phe concentration on the day of the start of 24-hr urine collection. Participants completed one dual X-ray absorptiometry (DXA) scan. The registered dietitian study coordinator maintained frequent contact in order to facilitate compliance with the protocol. Upon completion of each 24-h urine collection, samples were aliquoted and stored at −80°C.

### 2.3. Amino Acid and Glycomacropeptide Medical Foods

Baseline prescriptions for AA-MF or GMP-MF intake were provided by participants' home metabolic clinics. Participants consumed their preferred Phe-free AA-MF, which resulted in the use of 7 different AA-MF (Supplemental Table 1 in Supplementary Material, available online at https://doi.org/10.1155/2017/1909101). The main change in the glycomacropeptide treatment was the substitution of the AA-MF with the GMP-MF. The GMP-MF were donated by Cambrooke Therapeutics and contained Glytactin™, a proprietary formulation of ~70% glycomacropeptide (cGMP-20, Arla Foods Ingredients) and ~30% supplemental AAs (Arg, His, Leu, Trp, and Tyr). Participants recorded all nutritional intake for 48–72 h, starting 24–48 h prior to and during the urine collection. Total energy, macronutrient, micronutrients, and AAs were estimated based on food records using Food Processor SQL (Version 10.12.0, ESHA). We define natural foods as all food and beverages consumed that are not medical foods for PKU management.

### 2.4. Analytical Measurements

Urinary biomarker excretion concentrations were analyzed using standard techniques (LabCorp; Dublin, OH, USA). We measured renal net acid excretion (RNAE) directly using a validated research method [20, 21]. Titratable acid, bicarbonate, and net acid concentrations were obtained within one sample measurement. For each urine sample, pH was measured and an excess of HCl was added to the sample; the carbon dioxide formed from the reaction of the urine bicarbonate and HCl was driven off by boiling. The sample was then titrated back to the original pH of the urine sample with sodium hydroxide. The amount of bicarbonate was calculated as the difference in the sodium hydroxide volume added to the urine sample to reach the original urine pH and a water standard. The titration with the addition of sodium hydroxide continued to pH 7.4 to allow for measurement of titratable acid. The amount of titratable acid was calculated as the difference in the sodium hydroxide volume added to the urine sample from the original urine pH to pH 7.4 and a water standard. To determine RNAE, 8% formaldehyde was added to the sample and continued until the titration to pH 7.4 was reached. The amount of renal net acid was calculated as the difference in the sodium hydroxide volume added to the urine sample from pH 7.4 and a water standard. Concentration of NH_4_^+^ was calculated using the equation NH_4_^+^ = RNAE − titratable acid [20, 21].

Phe concentrations in dried blood spots were analyzed using the nonderivatized flow-injection analysis tandem mass spectrometry method [22]. Lumbar spine, dual femur, forearm, and total body bone mineral density (BMD) and body composition were measured using a single GE-Healthcare Lunar iDXA densitometer (Madison, WI, USA). All scans were measured and analyzed using enCORE software version 13.31 or 13.6. Weight-adjusted* Z*-scores were derived using the manufacturer's gender-specific normative database. Spine trabecular bone score (TBS), which is derived from the DXA image and provides information on bone texture and, therefore, serves as a microarchitecture surrogate and fracture risk factor independent of BMD, was obtained using Medimaps Group software version 2.0.0.1 or 2.1.0.0. (Mérignac, France) [23]. Appendicular lean mass (ALM) was calculated by adding the lean mass of both arms and legs, based on the DXA scan. Serum and plasma samples obtained during the clinical trial were analyzed using standard techniques in clinical laboratories. Cytokine concentrations were measured in plasma in duplicate or triplicate (intra-assay CV, 10–13%) using a Bio-Plex multiplex human cytokine assay kit (Biorad, M50-0KCAF0Y). Three separate cytokine determinations were obtained in 27 participants while consuming AA-MF over 8–12 weeks and one determination was obtained in participants after consuming GMP-MF for 3 weeks [17].

### 2.5. Statistical Analysis

All statistical analyses were performed using SAS version 9.4 and assumptions of normality and equal variance were tested. Most analyses for urinary biomarker excretion, nutrient intake, and blood Phe concentrations used PROC MIXED (SAS Institute Inc.). ANOVA was used to test for main effects for treatment, genotype (classical or variant PKU), and treatment-genotype interactions. When data was skewed, effects due to treatment or genotype were analyzed separately using the Kruskal-Wallis test. Most analyses for the serum chemistry profiles used PROC MIXED. ANOVA was used to test for main effects for treatment, sequence, and treatment-sequence interactions. Plasma cytokines were analyzed using PROC UNIVARIATE to compare sample median values with a general population median [24]. Correlations were calculated using Pearson's correlation coefficient. Statistical significance was set at* p* < 0.05.

## 3. Results

### 3.1. Determination of the Potential Renal Acid Load of Low-Phe Medical Foods

**Figure 1:**
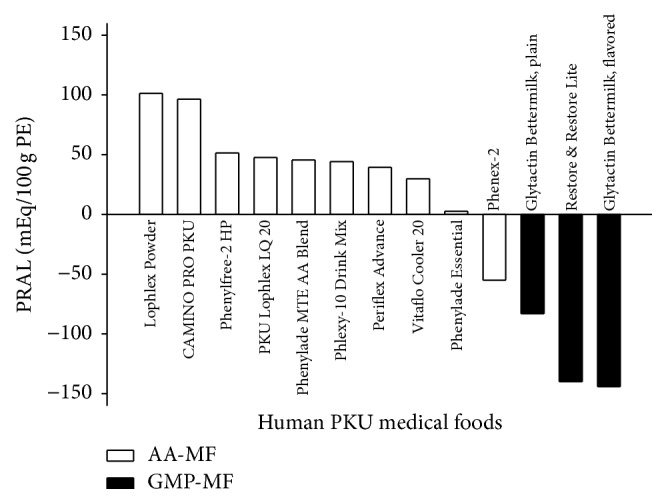
Potential renal acid load was calculated for 10 different AA-MF and 3 different GMP-MF, based on mineral and amino acid analysis,* n* = 2-3 per medical food. AA-MF, amino acid medical foods; GMP-MF, glycomacropeptide medical foods; PRAL, potential renal acid load; PE, protein equivalent.

In order to determine the dietary acid load, we analyzed mineral and AA content of 10 AA-MF and 3 GMP-MF and calculated the PRALs. Nine of the 10 AA-MF provided a 1.5- to 2.5-fold higher PRAL compared to the 3 GMP-MF (Figure 1). Thus, all 8 participants consumed AA-MF with a high-PRAL.

### 3.2. Characteristics of the Participants

The sample size of 8 participants (4 males and 4 females) included 2 minors, aged 16-17 y, and 6 adults, aged 19–35 y (Table 1). Four participants had classical PKU and four participants had a variant form of PKU [17, 25, 26]. Average blood Phe concentrations were not significantly different between treatments (mean ± SE, 401 ± 60 *µ*mol/L, for AA-MF compared with 469 ± 60 *µ*mol/L for GMP-MF;* p* = 0.15,* n* = 8). Three of 4 male participants and 2 of 4 female participants show excess body fat. All 8 participants demonstrate ALM/ht^2^ within normal limits; however, 4 participants were close to the minimum of the reference range [27]. Two participants had L1-4 and/or total body* Z*-scores ≤ −2.0, consistent with a clinical diagnosis of low BMD-for-age, and TBS values indicating partially degraded bone microarchitecture [28, 29].

### 3.3. Nutrient Profiles of the Diets

The nutrient profiles of the overall diets were generally constant, except that the source of PE was elemental AAs with AA-MF, and primarily intact protein with GMP-MF (Table 2). No significant differences were found in intake of total energy, total protein, and protein from medical foods (55–57 g protein from medical food/d).

The PRAL from medical foods was significantly higher with AA-MF compared to GMP-MF (means ± SE, −43 ± 6 mEq/d for GMP-MF compared to 39 ± 5 mEq/d for AA-MF, *p* < 0.0001), while PRAL from natural foods was not significantly different between treatments (−62 ± 11 mEq/d for GMP-MF compared to −54 ± 11 mEq/d for AA-MF,* p *= 0.44). Lower PRAL from medical foods with GMP-MF was primarily driven by not only significantly lower intakes of Cys (0.03 ± 0.01 g/d for GMP-MF compared to 2.5 ± 0.3 g/d for AA-MF, *p* < 0.0001), but also a significantly lower intake of Met from medical foods with GMP-MF (0.9 ± 0.1 g/d for GMP-MF compared to 1.3 ± 0.2 g/d for AA-MF,* p *= 0.047) and a significantly higher intake of sodium from medical foods with GMP-MF (Table 2). Arg and Lys are added to AA-MF as monohydrochloride forms and Arg monohydrochloride is added to GMP-MF; the chloride contributes to the PRAL calculation. Chloride intake from medical foods was higher with AA-MF (796 ± 220 for AA-MF compared to 563 ± 90 mg/d for GMP-MF;* p *= 0.290). Thus, the dietary acid load provided by medical foods rather than natural foods determined the higher dietary PRAL with the AA-MF treatment.

Intake of bone-related micronutrients (vitamins D, calcium, and phosphorus) surpassed the United States' Recommended Dietary Allowance or Adequate Intake guidelines for AA-MF and GMP-MF but was below the Tolerable Upper Intake Level (UL). For both treatments, magnesium intakes from medical foods (362–400 mg/d) were above the UL, which is defined as 350 mg/d magnesium from a pharmacological source. Additionally, participants had a significantly higher calcium-to-phosphorus (Ca : P) ratio, favorable for bone health [30] with GMP-MF (means ± SE, 1.22 ± 0.04 for GMP-MF compared to 0.96 ± 0.14 mg/d for AA-MF;* p *= 0.03).

### 3.4. Glycomacropeptide Medical Foods Reduce Urinary Excretion of Renal Net Acid, Calcium, and Magnesium

**Figure 2:**
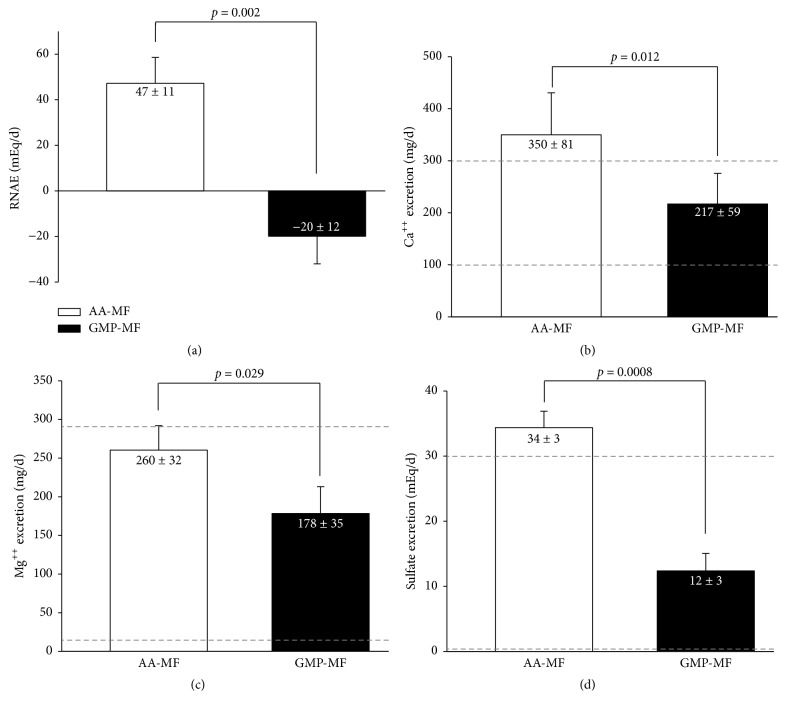
Renal net acid excretion (a), urinary calcium excretion (b), urinary magnesium excretion (c), and urinary sulfate excretion (d), based on 24-hr urine collections in participants with PKU who consumed AA-MF or GMP-MF,* n* = 8. Values are means ± SE. The dashed lines (b, c, d) represent the reference range for urinary calcium excretion (100–300 mg/d), magnesium excretion (12–293 mg/d), and urinary sulfate excretion (0–30 mEq/d). Results indicate significant increases in renal net acid excretion (*p *= 0.002), urinary calcium (*p* = 0.012), magnesium (*p *= 0.029), and sulfate (*p* = 0.0008) excretion with AA-MF compared to GMP-MF. AA-MF, amino acid medical foods; GMP-MF, glycomacropeptide medical foods.

**Figure 3:**
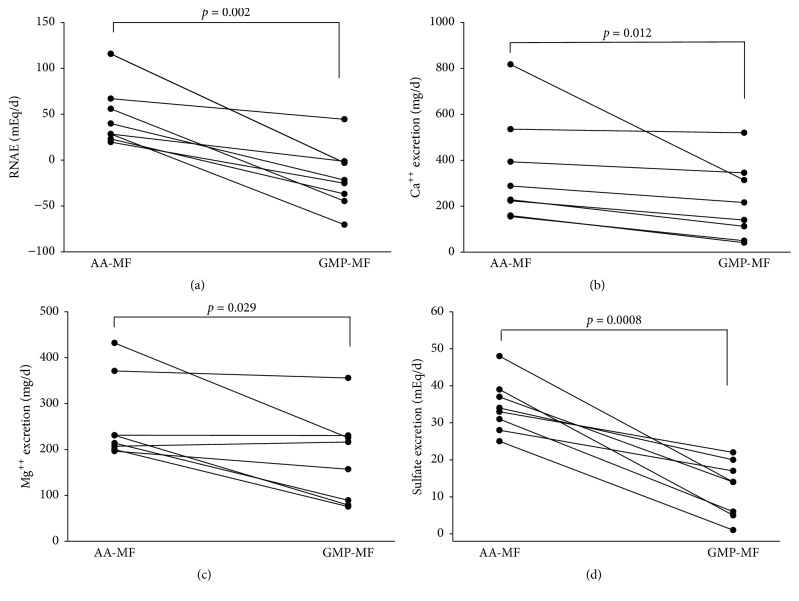
Renal net acid excretion (a), urinary calcium excretion (b), urinary magnesium excretion (c), and urinary sulfate excretion (d), based on 24-hr urine collections in participants with PKU who consumed AA-MF or GMP-MF,* n* = 8. Statistical significance reflects significant treatment effects, as shown in Figure 2. Results show increases among all 8 participants in urinary excretion of renal net acid, calcium, and sulfate and in 7 of 8 participants in urinary excretion of magnesium with AA-MF compared to GMP-MF. AA-MF, amino acid medical foods; GMP-MF, glycomacropeptide medical foods.

Consistent with the lower calculated PRAL, GMP-MF significantly reduced RNAE by over 3-fold (Figure 2(a)). The significant reduction in RNAE with GMP-MF was driven by significant decreases in urinary excretion of NH_4_^+^ and titratable acids (Table 3). Despite similar dietary intake of calcium and magnesium, 24-h urinary excretion of calcium and magnesium was significantly lower with GMP-MF compared with AA-MF (Figures 2(b) and 2(c)). Mean urinary calcium excretion was above the reference range and mean urinary magnesium was at the higher end of the reference range with AA-MF. Urinary excretion of sulfate was significantly higher with AA-MF compared to GMP-MF (Figure 2(d)), related to the significantly higher intakes of sulfur-containing AAs, Cys, and Met from AA-MF. Mean urinary sulfate was above the reference range with AA-MF, which indicates that the sulfur-containing AA consumption with AA-MF may be too high with potential relevance to excessive DNA methylation [31]. There were no significant main effects for genotype for the urinary excretion parameters. Presentation of individual participant data indicated a consistent response of higher RNAE, calcium, and sulfate excretion across all 8 participants and higher magnesium excretion in 7 of 8 participants with AA-MF compared to GMP-MF (Figure 3).

Urinary biomarkers were not corrected for urinary creatinine because urinary creatinine excretion was significantly higher during intake of AA-MF despite similar total urinary volume for both treatments (Table 3). In addition, hypersecretion of creatinine by proximal tubules with potential renal dysfunction [32–34] was noted in 5 of 8 participants, in spite of normal plasma electrolytes, blood urea nitrogen, and estimated glomerular filtration rate [17]. The average urinary total protein excretion was not significantly different for GMP-MF and AA-MF. Proteinuria, defined as >150 mg/d, was found in 3 participants, which is also suggestive of the potential for renal dysfunction. This provides additional support that correction for urinary creatinine is not appropriate.

**Figure 4:**
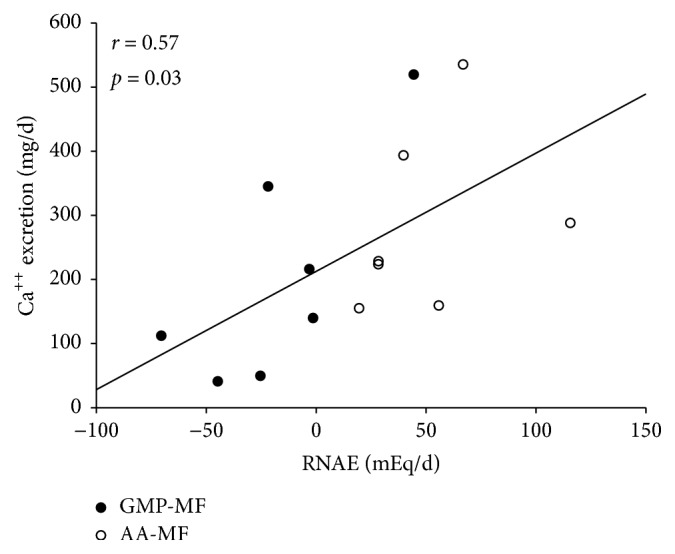
Urinary calcium and renal net acid excretion were positively correlated (*r* = 0.57,* p *= 0.03) with AA-MF and GMP-MF,* n* = 14 from 7 participants. One participant was omitted, due to use of a therapeutic dose of calcium to treat low BMD-for-age. AA-MF, amino acid medical foods; BMD, bone mineral density; GMP-MF, glycomacropeptide medical foods; RNAE, renal net acid excretion.

We used correlation coefficients to examine our hypothesis that intake of a high dietary acid load from AA-MF increases bone resorption and urinary calcium and magnesium, compared with a low dietary acid intake from GMP-MF. Urinary calcium (*r* = 0.57,* p *= 0.03,* n* = 14) (Figure 4) and urinary magnesium (*r* = 0.51;* p* = 0.045,* n* = 16) excretion were positively correlated with RNAE for both treatments, suggesting that RNAE is associated with increased urinary calcium and magnesium excretion, possibly from increased bone resorption.

Participants had lifelong AA-MF intake at the time of urine collection, whereas consumption of GMP-MF was short-term. Interestingly, urinary calcium was negatively correlated with L1–L4 BMD (g/cm^2^) while consuming AA-MF (*r* = −0.79,* p *= 0.02;* n* = 8) but not while consuming GMP-MF (*r* = −0.48,* p *= 0.31;* n* = 8). Given the lifelong intake of AA-MF, these results suggest that urinary calcium excretion with AA-MF is associated with a lower L1–L4 BMD. Intake of protein equivalents from AA-MF was negatively correlated with total body BMD (g/cm^2^) (*r* = −0.77,* p *= 0.04;* n* = 7), but not L1–L4 BMD.

Blood Phe concentrations were not significantly correlated with urinary calcium (*r* = 0.09;* p *= 0.74;* n* = 16), magnesium (*r* = −0.37;* p *= 0.16;* n* = 16), or RNAE (*r* = 0.01;* p *= 0.97;* n* = 16) for both treatments. This indicates that blood Phe concentrations were not associated with urinary excretion of bone-related minerals.

### 3.5. Bone-Related Biomarkers

Average serum concentrations of 25(OH)D, Bone-Specific Alkaline Phosphatase (BSAP), N-telopeptide (NTX), and serum calcium were within normal limits for both treatments (Table 3). When participants were separated by gender, average BSAP concentrations are above the reference range (6.5–20.1 *µ*g/L) with AA-MF and GMP-MF for males, but not females. Average NTX concentrations were within the gender-specific reference ranges during both treatments. Carbon dioxide was significantly higher with GMP-MF compared to AA-MF [17].

### 3.6. Plasma Cytokines

Concentrations of cytokines in plasma were determined given the evidence that a GMP diet decreases cytokines in mice relative to an AA diet [8, 35] and the known link between increased bone resorption due to inflammation [36]. With consumption of AA-MF, we observed significantly higher levels of the bone-related inflammatory cytokines, IL-1*β*, IL-17, TNF-*α*, IFN-*γ*, and IL-6, in our participants with PKU (*n* = 27) compared to the general population median reference values [24] (Table 4). Similar to AA-MF, plasma cytokines were also elevated during consumption of GMP-MF (data not shown).

## 4. Discussion

Skeletal fragility is a complication of PKU that has not been investigated in controlled intervention studies. We hypothesized that skeletal fragility partly relates to the high dietary acid load provided by AA-MF. Our hypothesis is supported by evidence that chronic consumption of a high dietary acid load evokes skeletal buffering of H^+^ to maintain neutral pH, increasing bone resorption and renal calcium excretion [13]. In this pilot study, we found that current AA-MF provided 1.5- to 2.5-fold higher PRAL compared to GMP-MF. Additionally, consumption of AA-MF significantly increased urinary excretion of renal net acid (3-fold ↑), calcium (40% ↑), and magnesium (30% ↑) compared with GMP-MF. Although bone breakdown and calcium balance were not directly measured in this study, four randomized, double-blind, placebo-controlled trials confirm that neutralizing dietary acid load with potassium citrate [14, 15] or potassium bicarbonate [37] reduces renal calcium excretion and bone resorption, increasing calcium balance [14] and improving BMD [15].

Low BMD-for-age in premenopausal women and men < 50 y, is defined as 2 standard deviations below the mean (*Z*-score ≤ −2.0) [16, 28]. The incidence of low BMD in the general population is 2%. In contrast, up to 20% of individuals with PKU show low BMD with* Z*-scores ≤ −2.0 [5, 7], and in this study 2 of 8 participants had low BMD-for-age and evidence of bone microarchitectural degradation. Factors contributing to low BMD are the PKU genotype and the low-Phe diet, restricted in natural foods with provision of most nutrients from synthetic AA-MF. Our murine studies demonstrated reductions in bone size and strength in PKU (*Pah*^enu2^) and wild type mice fed diets containing a high dietary acid load and synthetic AAs compared with increased bone size and strength when mice consumed diets with a lower dietary acid load and intact protein from casein or GMP [9]. Similarly, we demonstrated in human PKU that AA-MF have a high dietary load compared to GMP-MF and may negatively affect bone health by increasing urinary excretion of renal net acid, calcium, and magnesium. Supporting our hypothesis, a recent study using mathematical modeling also found that AA-MF intake was negatively correlated with total body BMD in PKU patients [38]. In summary, our study suggests that compensation for a high dietary acid with increased excretion of renal acid and minerals may contribute to low BMD and microarchitectural deterioration in PKU.

Controversy exists regarding evidence that a high dietary acid load contributes to low bone mass via skeletal buffering, increased bone resorption, and calciuria [15]. In older adults (aged > 55 y) with age-related declines in renal function, researchers demonstrated adverse effects of a high dietary acid load on calcium balance [14] and BMD [15]. These studies in older adults may apply to younger populations with PKU, who may also have impaired renal function due to consumption of AAs as a primary protein source [12]. Indeed, Hennerman et al. reported impaired renal function in 19% of patients with PKU and observed declining glomerular filtration rates and increasing urinary calcium excretion corresponding with graduated increases in AA intake (0, 0.70, and 0.94 g PE/kg body weight). The skeleton and kidneys help regulate acid-base homeostasis, yet the lifelong management of PKU with synthetic AAs is only now being recognized as harmful to renal and skeletal health.

Our study provides further insights into three factors contributing to bone health in PKU beyond the higher dietary acid load provided by AA-MF. First, elevated inflammatory cytokines in individuals with PKU likely contribute to bone loss and microarchitectural deterioration because inflammatory cytokines trigger osteoclast activation and bone resorption [36]. This study provides the first evidence of bone texture alteration in PKU using the TBS. Cytokines related to bone metabolism were elevated in our participants and were not significantly different between AA-MF and GMP-MF treatments. This lack of difference may reflect the 3-wk GMP-MF treatment being too short and/or the PKU genotype as the predominant driver of elevated cytokine concentrations. Second, bone-muscle functional interactions demonstrate that reduced lean mass or muscular abnormalities can contribute to bone resorption, reduced bone strength, and increased risk for fracture [6, 18]. For example, in healthy young adults at bedrest, a high dietary acid load induced by AA supplementation increased urinary markers of bone resorption (NTX and deoxypyridinoline) and calcium excretion [18]. Consistent with the low-normal ALM/ht^2^ found in 4 of our participants, a recent study in patients with PKU using peripheral quantitative computed tomography showed reduced BMD and lower bone strength in relation to muscular force in the radius [6]. Third, the bioavailability of minerals in medical foods used to manage PKU is unknown and might improve with GMP, which demonstrates prebiotic properties [35, 39]. Additionally, the Ca : P ratio in our participants was low with AA-MF (<1.0) and optimal with GMP-MF (~1.22), which may impact mineral bioavailability and bone resorption [30].

Evidence demonstrates that blood Phe concentrations are not significant contributors to the bone phenotype in PKU [4, 5, 7, 38]. Our data are consistent with cross-sectional studies that show no correlation between blood Phe concentrations and BMD. Moreover, in cultured human primary osteoblasts, we found no association between mineralization and increasing Phe concentrations of 0, 200, 600, and 1200 *µ*mol/L (unpublished data).

Strengths of our study include a crossover design that was balanced for gender and PKU genotype and statistical significance despite a small sample size. Limitations of our study include a short GMP dietary treatment of 1–3 wks and inclusion of 7 different AA-MF to allow for participant preference.

## 5. Conclusions

This is the first human clinical trial comparing the effects of medical foods with differing dietary protein sources and acid loads on urinary excretion of renal net acid and minerals in PKU. We established that AA-MF provided a high dietary acid load, relative to Glytactin GMP-MF. The high dietary acid load increased urinary excretion of renal net acid, calcium, magnesium, and sulfate, likely contributing to the etiology of skeletal fragility in PKU. Future controlled, human intervention studies addressing the relationships among dietary acid load, medical food protein source, and mineral balance on bone status in PKU are needed [16].

## Supplementary Material

Supplemental Table 1 provides a summary of the preferred PKU AA-MF and GMP-MF, including the brand name, company affiliation, form (i.e., powder, liquid etc.) and the number of participants that consumed each product. Among the 8 participants enrolled in this pilot study, 8 different AA-MF from multiple companies, including Nutricia, Cambrooke Therapeutics and Mead Johnson were consumed, while 7 different GMP-MF products from Cambrooke Therapeutics were consumed.

## Figures and Tables

**Table 1:** Characteristics of enrolled participants (*n* = 8).

	Males				Females			
*Participant number*	1	2	3	4	5	6	7	8
*Age*	34	34	19	17	35	35	28	16
*Classical/variant* ^*2*^	Classical	Variant	Classical	Variant	Variant	Classical	Classical	Variant
*Mutation* ^*4*^	IVS1nt5G>T; IVS12nt1G>A	R408W; IVS12nt1G>A	R408W; Y356X	p.R68S; IVS12+1G>T	E280K; E390G	L242F; R408W	p.F55>Lfs; R408W	p.R157N; p.L348V
*Total body*								
Total fat mass, %	35	34	36	11	42	39	26	30
ALM/ht^2^, kg/m^2^	9.27	7.41	7.80	7.28	7.49	7.17	5.95	6.54
BMD, g/cm^2^	1.131	0.938	1.113	1.078	1.233	1.077	1.035	1.166
*Z*-score	−1.6	−2.3	−0.7	−1.0	1.0	−0.3	0.0	0.8
*Spine L1–L4*								
BMD, g/cm^2^	1.026	0.800	1.145	1.134	1.375	1.059	1.114	1.106
*Z*-score	−2.4	−3.3	−0.6	−0.6	1.2	−1.2	−0.2	−0.6
Trabecular bone score	1.273	1.233	1.389	1.508	1.420	1.320	1.490	1.425
*Femoral neck*								
BMD, g/cm^2^	0.925	X^1^	1.020	1.075	X	1.008	0.973^2^	1.025
*Z*-score	−1.5	X	−0.7	−0.3	X	−0.1	−0.1^2^	0.2
*Femoral trochanter*								
BMD, g/cm^2^	0.731	X	0.745	0.989	X	0.811	0.852^2^	0.962
*Z*-score	−2.4	X	−1.8	0.3	X	−0.3	0.4^2^	1.4
*Total dual femur*								
BMD, g/cm^2^	0.896	X	0.999	1.135	X	1.080	1.081^2^	1.134
*Z*-score	−1.8	X	−0.9	0.1	X	0.6	0.9^2^	0.9
*Radius 33%*								
BMD, g/cm^2^	0.892	X	0.982	0.730	X	0.812	0.779	0.838
*Z*-score	−1.0	X	N/A^3^	N/A^3^	X	−0.7	−1.1	N/A^3^

^1^Symbol represents data that was unable to be obtained at the time of DXA scan completion.

^2^Femoral DXA parameters for one participant are based on the right femur only due to presence of metal in the left hip.

^3^*Z*-Scores were unable to be calculated for 3 participants because reference population data for the 33% radius for individuals < 20 y were not in the GE-Healthcare Lunar database. AA-MF, amino acid medical food; ALM, appendicular lean mass; BMD, bone mineral density; DXA, dual X-ray absorptiometry; Spine L1–4, Spine Lumbar 1–4.

**Table 2:** Nutrient profiles of the low-Phe diet in combination with AA-MF and GMP-MF^1^.

	AA-MF	GMP-MF	*p*
*Energy*			
kcal/d	2,266 ± 263	2,566 ± 204	0.26
kcal from MF/d	544 ± 100	763 ± 118	0.049
kcal from NF/d	1,722 ± 229	1,802 ± 127	0.77
*Protein*			
g protein/d	79 ± 4	81 ± 6	0.70
g protein from MF/d	57 ± 5	55 ± 7	0.72
g protein from NF/d	21 ± 2	26 ± 4	0.32
*Calcium : phosphorus ratio* ^*3*^			
Ca : P ratio/d	1.06 ± 0.14	1.19 ± 0.17	0.13
Ca : P ratio from MF/d	0.96 ± 0.14	1.22 ± 0.04	0.03
Ca : P ratio from NF/d	0.90 ± 0.27	1.38 ± 0.66	0.28
*Vitamin D* ^*2*^			
IU vitamin D/d	630 ± 230	680 ± 167	0.75
IU vitamin D from MF/d	623 ± 182	593 ± 131	0.82
IU vitamin D from NF/d	54 ± 30	60 ± 26	0.81
*Calcium* ^*2*^			
mg calcium/d	1,745 ± 274	1,898 ± 270	0.69
mg calcium from MF/d	1,282 ± 240	1,408 ± 264	0.58
mg calcium from NF/d^3^	416 ± 78	484 ± 76	0.39
*Magnesium*			
mg magnesium/d	568 ± 62	684 ± 113	0.27
mg magnesium from MF/d	362 ± 68	400 ± 68	0.43
mg magnesium from NF/d	206 ± 30	284 ± 77	0.37
*Phosphorus*			
mg phosphorus/d	1,836 ± 228	1,749 ± 161	0.62
mg phosphorus from MF/d	1,183 ± 229	1,131 ± 195	0.80
mg phosphorus from NF/d	653 ± 58	618 ± 69	0.70
*Potassium*			
mg potassium/d	3,249 ± 403	3,699 ± 383	0.40
mg potassium from MF/d	1,100 ± 307	1,388 ± 175	0.39
mg potassium from NF/d	2,149 ± 324	2,311 ± 302	0.70
*Sodium*			
mg sodium/d^2^	2,559 ± 298	3,487 ± 334	0.006
mg sodium from MF/d	499 ± 123	1,251 ± 176	0.017
mg sodium from NF/d^2^	2,060 ± 271	2,236 ± 270	0.48

^1^Values are means ± SE, based on 24-h food records, *n* = 8.

^2^Vitamin D and calcium intake is based on 7 participants. One participant was omitted due to use of a therapeutic dose of Vitamin D and calcium throughout the study, prescribed for low BMD-for-age.

^3^Calcium intake from natural foods had a significant genotype effect (*p* = 0.02). Participants with classical PKU consumed less calcium compared to those with variant PKU (means ± SE, 327 ± 48 mg calcium/d with classical PKU compared to 614 ± 57 mg calcium/d with variant PKU, *p* = 0.02). AA-MF: amino acid medical foods, GMP-MF: glycomacropeptide medical food, MF: medical foods, NF: natural food, and PRAL: potential renal acid load.

**Table 3:** Bone-related blood and urine biomarkers with AA-MF or GMP-MF^1^.

Test	*n*	AA-MF	GMP-MF	*p*
*Blood Biomarkers* ^*2*^				
Vitamin D, 1,25(OH)_2_D, pg/mL	19	65.4 ± 3.39	71.9 ± 4.10	0.079
Vitamin D, 25(OH)D, ng/mL	28	33.6 ± 1.53	33.8 ± 1.70	0.797
Calcium, mg/dL	30	9.07 ± 0.07	9.10 ± 0.07	0.706
BSAP, *µ*g/L	26	17.0 ± 2.20	17.0 ± 2.08	0.966
BSAP (females), *µ*g/L	18	12.9 ± 1.08	13.6 ± 1.37	0.455
BSAP (males), *µ*g/L	9	25.2 ± 5.42	23.8 ± 5.05	0.416
NTX, nmol/L BCE	27	17.1 ± 0.65	17.5 ± 0.66	0.629
NTX (females), nmol/L BCE	18	14.7 ± 1.94	15.0 ± 1.48	0.345
NTX (males), nmol/L BCE	9	21.1 ± 1.98	18.4 ± 2.56	0.370
*Urinary biomarkers* ^*3*^				
Basic				
Volume, L/d	8	1.74 ± 0.22	1.66 ± 0.16	0.88
Creatinine, mg/d	8	2921 ± 497	1987 ± 417	0.02
Total protein, mg/d	8	129 ± 21	151 ± 32	0.47
Specific gravity	8	1.016 ± 0.002	1.018 ± 0.002	0.38
Titration				
Renal net acid excretion, mEq/d	8	47 ± 11	−20 ± 12	0.002
Ammonium (NH_4_^+^), mmol/d	8	44 ± 6	16 ± 5	0.0007
Titratable acid, mmol/d	8	3 ± 7	−36 ± 8	0.005
Mineral excretion				
Chloride, mEq/d	8	295 ± 50	212 ± 51	0.052
Calcium, mg/d	8	350 ± 81	217 ± 59	0.01
Magnesium, mg/d	8	260 ± 32	178 ± 35	0.03
Phosphorus, mg/d	8	1478 ± 281	1293 ± 366	0.08
Potassium, mEq/d	8	126 ± 20	112 ± 20	0.49
Sodium, mEq/d	8	266 ± 44	210 ± 33	0.22
Sulfate, mEq/d	8	34 ± 3	12 ± 3	0.0008

^1^Values are means ± SE. The *p *values included in this table represent the treatment comparisons.

^2^Results are based on fasting venipunctures obtained during the clinical trial with AA-MF or GMP-MF [17]. Vitamin D and bone turnover markers were measured in serum and calcium was measured in plasma. Statistical analysis included ANOVA with effects for treatment, baseline, sequence, and treatment-sequence interaction. NTX was analyzed by ANCOVA with baseline levels as a covariate. For all other tests, baseline was not significantly different.

^3^Results are based on 24-hr urine collections with AA-MF or GMP-MF. Statistical analysis included ANOVA with effects for treatment, genotype, and treatment-genotype interaction. There were no significant genotype effects for urinary biomarker excretion comparisons. AA-MF, amino acid medical foods; GMP-MF, glycomacropeptide medical foods; BSAP, bone-specific alkaline phosphatase; NTX, N-terminal telopeptide.

**Table 4:** Plasma cytokine concentrations in participants with phenylketonuria consuming AA-MF compared to a general population.

Cytokine, pg/mL	Comparison group		*p*
	Phenylketonuria^1^	General population^2^	
IL-1*β*	3.2 (2.5–3.4)	2.6 (0.8–3.9)	0.0085
IL-17	27 (14–35)	21.1 (6.5–38.5)	0.0411
TNF-*α*	40 (30–46)	35.3 (14.2–61.7)	0.0359
IL-12	8.2 (5.6–12.1)	15.8 (8.6–27.2)	<0.0001
IFN-*γ*	93 (78–110)	77.1 (48.4–127.6)	0.0002
IL-6	7.7 (6.2–9.3)	4.6 (1.1–10.8)	<0.0001
IL-10	10 (7.2–15)	4.3 (2.4–6.6)	<0.0001

^1^Values are the median (25th–75th quartile), based on 3 determinations over 8–12 wks in participants with phenylketonuria consuming AA-MF (*n* = 27). Statistical analysis was performed using PROC UNIVARIATE to compare sample median values with the population median.

^2^Population median reference values based on male and female participants, *n* = 254 [24]. AA-MF, amino acid medical foods.

## Abbreviations

AA: Amino acid
ALM: Appendicular lean mass
AA-MF: Amino acid medical foods
BMD: Bone mineral density
BSAP: Bone-specific alkaline phosphatase
DXA: Dual-energy X-ray absorptiometry
Ca : P: Calcium-to-phosphorus ratio
GMP-MF: Glycomacropeptide medical foods
NTX: N-terminal telopeptide
PAH: Phenylalanine hydroxylase
PE: Protein equivalent
PRAL: Potential renal acid load
RNAE: Renal net acid excretion
TBS: Trabecular bone score
UL: Tolerable upper intake level.
