# Supplementary material for: Amino Acid Medical Foods Provide a High Dietary Acid Load and Increase Urinary Excretion of Renal Net Acid, Calcium, and Magnesium Compared with Glycomacropeptide Medical Foods in Phenylketonuria
Source: J Nutr Metab. 2017 May 4;2017:1909101. doi: 10.1155/2017/1909101 (PMC5436062; doi:10.1155/2017/1909101)
Supplement: Supplementary file 1 — Supplemental Table 1 provides a summary of the preferred PKU AA-MF and GMP-MF, including the brand name, company affiliation, form (i.e., powder, liquid etc.) and the number of participants that consumed each product. Among the 8 participants enrolled in this pilot study, 8 different AA-MF from multiple companies, including Nutricia, Cambrooke Therapeutics and Mead Johnson were consumed, while 7 different GMP-MF products from Cambrooke Therapeutics were consumed. [file 1909101.f1.docx]

SUPPLEMENTAL TABLE 1: Brand descriptions of medical foods used by participants^1^.

| **AA-MFs** | | | |
| --- | --- | --- | --- |
| **Brand name** | **Company^2^** | **Form^3^** | **Number of Subjects^4^** |
| Phenyl-free 2 HP | Mead Johnson Nutrition | powder, can | 2 |
| CAMINO PRO PKU | Cambrooke Therapeutics | liquid, RTS | 1 |
| Lophlex Powder | Nutricia | powder, can or pre-portioned packet | 1 |
| Periflex Advance | Nutricia | powder, can | 1 |
| PhenylAde Essential Drink Mix | Nutricia | powder, can | 1 |
| PhenylAde MTE Amino Acid Blend | Nutricia | powder, can | 1 |
| Phlexy-10 Tablets | Nutricia | pills | 1 |
|  |  |  |  |
| **GMP-MFs** | | | |
| **Brand name** | **Company** | **Form** | **Number of Subjects** |
| Glytactin Bettermilk | Cambrooke Therapeutics | powder, pre-portioned packet | 5 |
| Glytactin COMPLETE 15 | Cambrooke Therapeutics | meal replacement bar, RTS | 4 |
| Glytactin RESTORE LITE | Cambrooke Therapeutics | liquid, RTS | 3 |
| CaminoPro Pudding with Glytactin^5^ | Cambrooke Therapeutics | powder, pre-portioned packet | 3 |
| Glytactin RTD 15^6^ | Cambrooke Therapeutics | liquid, RTS | 2 |
| Glytactin RESTORE | Cambrooke Therapeutics | liquid, RTS | 1 |
| Glytactin SWIRL Caramel | Cambrooke Therapeutics | powder, pre-portioned packet | 1 |
| ^1^ AA medical food use was based on subject preference and their medical food prescription, which resulted in the use of 7 different AA medical foods. Subjects often used more than 1 medical food during the AA-MF and GMP-MF treatments.  ^2^The company represents product ownership when the study ended in 2015.  ^3^ The form of the medical food and the preparation required varied. Medical foods that were in liquid or solid bar form and RTS required no measuring or mixing. Medical foods that were in a powder form in a can required measurement and mixing, while medical foods that were in powder form in pre-portioned in commercial packets required mixing only.  ^4^Number of subjects taking each medical food is indicated.  ^5^The commercially availability of CaminoPro Pudding with Glytactin was discontinued prior to study completion of 8 subjects. ^6^ Glytactin RTD 15 became commercially available in 2014, which was within the last year of study completion. AA-MFs, amino acid medical foods; GMP-MFs, glycomacropeptide medical foods; HP, high protein; PKU, phenylketonuria; RTS, ready-to-serve. | | | |
